# Establishing Diagnostic Reference Levels for Single-Photon Emission Computed Tomography (SPECT) Imaging: A Preliminary Study in Indian Healthcare Settings

**DOI:** 10.7759/cureus.71740

**Published:** 2024-10-17

**Authors:** Ashutosh Pandey, Vandana K Dhingra, Mayank Goswami, Prabhaker Mishra, Pankaj Sharma

**Affiliations:** 1 Nuclear Medicine, All India Institute of Medical Sciences, Rishikesh, Rishikesh, IND; 2 Physics, Indian Institute of Technology (IIT) Roorkee, Roorkee, IND; 3 Biostatistics and Health Informatics, Sanjay Gandhi Postgraduate Institute of Medical Sciences, Lucknow, IND; 4 Radiology, All India Institute of Medical Sciences, Rishikesh, Rishikesh, IND

**Keywords:** diagnostic reference level, institutional reference level, nuclear medicine, radiation dose, single photon emission computed tomography

## Abstract

Introduction

In nuclear medicine, there is a critical need to balance the control and optimization of radiation doses to ensure patient safety while achieving high-quality diagnostic outcomes. However, diagnostic reference levels (DRLs) for nuclear medicine imaging studies have not been established in India. This study aims to fill this gap by establishing institutional DRLs and assessing radiation dose data specifically for Nuclear Medicine SPECT examinations.

Methods

This research was a cross-sectional observational study conducted in a single tertiary care setting, involving 1,250 patients. Data on the administered activity for 18 common single photon emission computed tomography (SPECT) procedures were gathered from 2019 to 2021. The 75th percentile (Q3) was established as the baseline for the institutional DRLs. Furthermore, the study conducted comparative analyses with established international DRLs.

Results

The study proposed institutional DRLs based on 18 SPECT nuclear medicine imaging examinations. The highest Q3 DRLs were identified for 99mTc-sestamibi (1193 MBq), followed by 99mTc-methylene diphosphonate (747 MBq) and 99mTc-ethyl cysteinate dimer (608 MBq). The lowest DRL was recorded for 99mTc gastric emptying imaging (37 MBq). Comparisons with international DRLs indicated close alignment with European Association of Nuclear Medicine Medical Internal Radiation Dose guidelines, while DRLs set by the National Council on Radiation Protection and Measurements (NCRP) were identified as distinct in several key aspects when compared to other established DRLs.

Conclusion

This research marks a significant step toward standardizing SPECT procedure doses in India, contributing to improved patient safety and care in nuclear medicine. The established DRLs will enhance the optimization of administered activities, ensure adherence to international standards, and improve diagnostic quality while reducing radiation exposure. These institutional DRLs will serve as the foundation for developing a roadmap to establish national DRLs.

## Introduction

As per the World Nuclear Association's 2024 reports, over 50 million nuclear procedures are conducted annually, with the demand for radioisotopes rising each year. According to a report published by the Atomic Energy Regulatory Board (AERB) in 2022, nuclear medicine procedures in India have been steadily increasing. As this growth persists, there has been a significant increase in medical radiation exposure for patients, family members, healthcare professionals, and the environment [1]. Although there are no restrictions on patient exposure, it should be reduced to the lowest level technically feasible, considering economic and social factors. Regular monitoring and evaluation of occupational and patient doses are essential to curtail potential radiation risks, including the potential for carcinogenesis [2]. Establishing diagnostic reference levels (DRLs) is a crucial tool for reducing unnecessary medical radiation exposure and addressing social concerns, as well as for optimizing radiation use in imaging. It serves as a reference level for an easily measurable quantity, typically the administered activity in megabecquerels (MBq) in nuclear medicine imaging studies [3]. A common misconception is that the DRL signifies a recommended activity level or a limit, suggesting that higher activities should not be administered. This misunderstanding may arise from variations in the definition of DRL across different guiding documents [4,5]. The earlier European Commission (EC) publication [4] introduced a recommended activity level, while the latest International Commission on Radiological Protection (ICRP) publication suggests using a reference level of doses based on an analysis of local or institutional practices [5]. In India, the first nuclear medicine DRLs for six radiopharmaceuticals were published in the Indian Council of Medical Research (ICMR) bulletin [6]; however, there is still a lack of implementation of the DRL approach in nuclear medicine procedures. Therefore, the goal was to establish the first institutional DRLs for administered activity in single-photon emission computed tomography (SPECT) imaging procedures with ICRP recommendations [5]. This research outlines the process of establishing the first institutional DRLs for SPECT procedures in India. Dose distribution data from various SPECT imaging procedures was analyzed to calculate the 75th percentile (Q3) for establishing the DRLs. Additionally, we compared our findings with those from various international organizations and countries.

## Materials and methods

Study design

This research was a single-center, cross-sectional observational study conducted at the All India Institute of Medical Sciences in Rishikesh, India. Data on administered activity (MBq) for radioisotopes 99mTc and I-131 in SPECT procedures were collected from 2019 to 2021. Ethical clearance was obtained from the Institutional Ethics Committee (IEC code: 47/IEC/PhD/2018). The study included 18 common nuclear medicine SPECT procedures: bone imaging, brain imaging, gastric emptying imaging, liver imaging, lung perfusion imaging, lymphoscintigraphy, Meckel's imaging, myocardial perfusion (rest and stress), renal diethylenetriamine pentaacetate (DTPA), renal ethylene dicysteine (EC), renal dimercaptosuccinic acid (DMSA), parathyroid imaging, thyroid imaging, salivary gland imaging, whole-body Iodine (I-131) thyroid imaging, and testicular imaging. Both male and female participants were included to establish institutional DRLs, while pregnant and breastfeeding women were excluded from the study. Equipment details are mentioned in Table 1.

**Table 1:** SPECT/CT equipment details. SPECT: Single-Photon Emission Computed Tomography.

Maximum number of slices	16
Tube current	10-440 mA
Tube voltage	80,100,120,140 kVp
Scan times	1920 slices/min
Manufacturer name	GE Healthcare Technologies Inc.
Model	GE Discovery NM/CT 670
Mode	Dual head variable angle

Data collection

Data on administered activity in SPECT/CT nuclear medicine imaging studies (GE Discovery NM/CT670) were collected from 1,250 cases using clinical history sheets from 2019 to 2021 and recorded in Microsoft Excel. Only examinations with complete patient information, age, gender, date, dose information, and study description, were included. The study documented the types of exams and the average administered radiopharmaceutical doses for each procedure performed at the institution. Before data collection, equipment calibration and quality control procedures were verified. Incomplete data and missing values were removed.

Statistical analysis

Complete data were analyzed and tabulated using R software (The R Foundation ©, Vienna, Austria) to calculate descriptive statistics, including range, mean, median, mode, interquartile range (Q1, Q2, Q3), and SD. Institutional DRLs were then compared with existing DRLs from other countries and organizations using box plots and heat maps.

## Results

Table 2 presents the established institutional DRLs for various imaging procedures, detailing the associated radiopharmaceuticals and their minimum and maximum ranges. It also includes the mean, standard deviation, and the 25th, 50th, and 75th percentiles. To determine the institutional DRLs, we focused on the 75th percentile values across all procedures. The highest 75th percentile values were noted for 99mTc-sestamibi (MIBI) (1193 MBq), 99mTc-methylene diphosphonate (MDP) (747 MBq), and 99mTc-ethyl cysteinate dimer (ECD) (608 MBq), while the lowest was found for 99mTc gastric emptying imaging (37 MBq).

**Table 2:** DRLs for SPECT/CT nuclear medicine imaging procedures. SPECT: Single-Photon Emission Computed Tomography; DRL: Diagnostic Reference Level.

Imaging Procedure	Radiopharmaceuticals	Min	Max	Percentile			Mean	SD	DRLs
				25th	50th	75th			
Bone Imaging	99mTc MDP	532	939	674	721	747	707.08	71.82	747
Brain Imaging	99mTc ECD	355	610	402	573	608	528.17	119.1	608
Gastric Emptying Imaging	99mTc Sulphur colloid	37	51.8	37	37	37	38.92	4.03	37
Liver Imaging	99mTc HIDA	144	188	155	170	177	167.29	13.16	177
Lung Perfusion Imaging	99mTc MAA	151	200	157	174	197	175.93	19.84	197
Lymphoscintigraphy	99mTc Nano-colloid	37	52	38	44	50	44.4	5.85	50
Meckel’s Imaging	99mTc pertechnetate	181	211	181	192	205	193.14	12.65	205
Myocardial Perfusion, stress 1 day	99mTc MIBI	309	377	325	342	357	343.86	20.52	357
Myocardial Perfusion, rest 1 day	99mTc MIBI	476	876	521	577	684	601.27	104.9	684
Myocardial Perfusion, stress- rest 1 day	99mTC MIBI	825	1309	1004	1121	1193	1093.9	145.5	1193
Renal Imaging	99m Tc EC	88	181	133	148	156	142.33	20.42	156
Renal Imaging	99m Tc DTPA	85	188	146	160	170	155.58	22.07	170
Renal Imaging	99m Tc DMSA	77	188	160	170	181	159.65	36.29	181
Parathyroid Imaging	99mTc MIBI	392	469	429	455	465	444.20	25.31	465
Thyroid Imaging	99m Tc pertechnetate	66	192	129	155	166	146.93	30.68	166
Salivary Gland Imaging	99m Tc pertechnetate	144	185	148	152	178	161.39	16.87	178
Whole body I-131 Thyroid Imaging	131 I	159	410	179	207	327	247.9	86.43	327
Testicular Imaging	99m Tc pertechnetate	144	196	155	175	192	172.05	18.84	192

**Figure 1:**
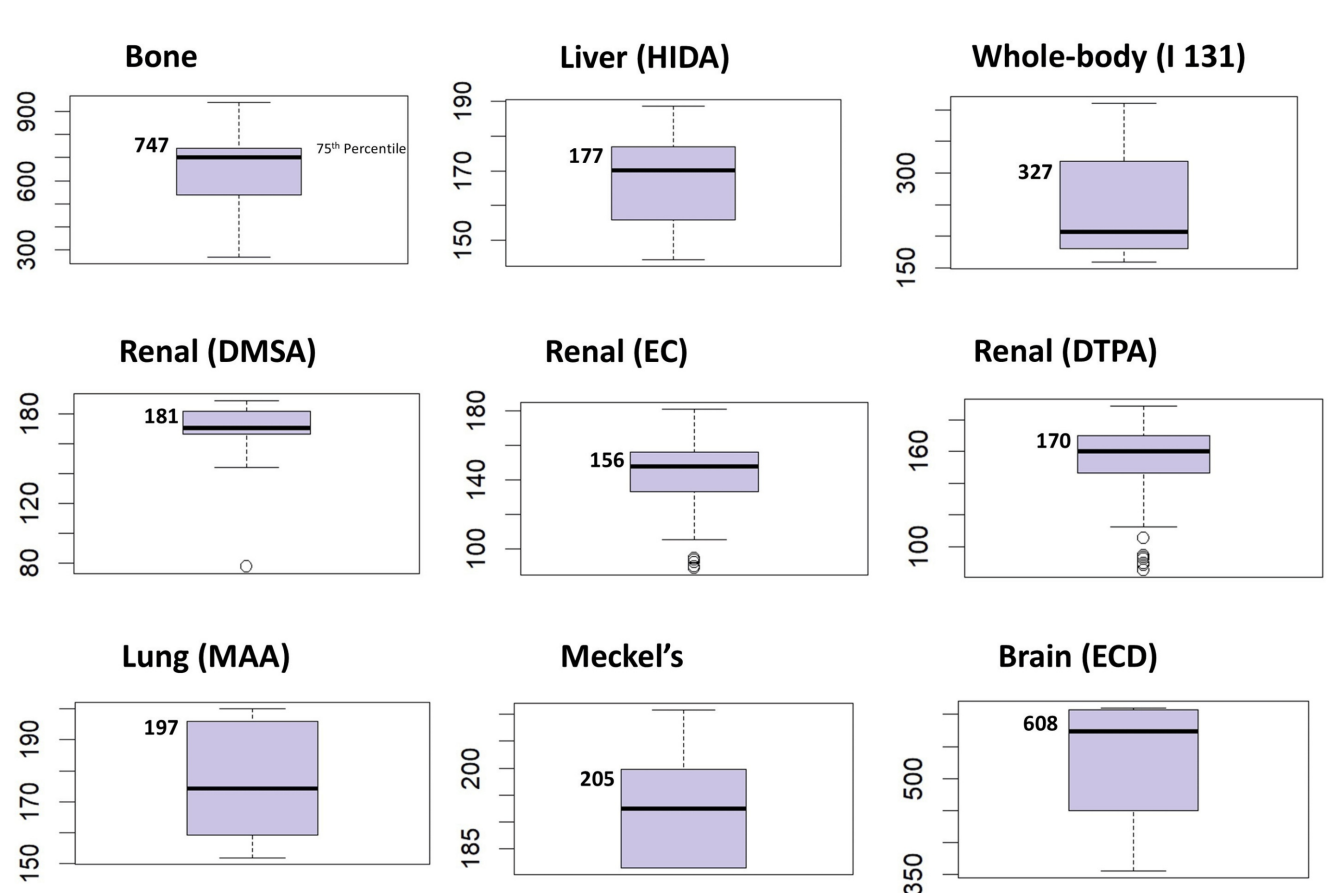
Distribution of institutional DRLs interquartile range showing 75th percentile. DRL: Diagnostic Reference Level.

**Figure 2:**
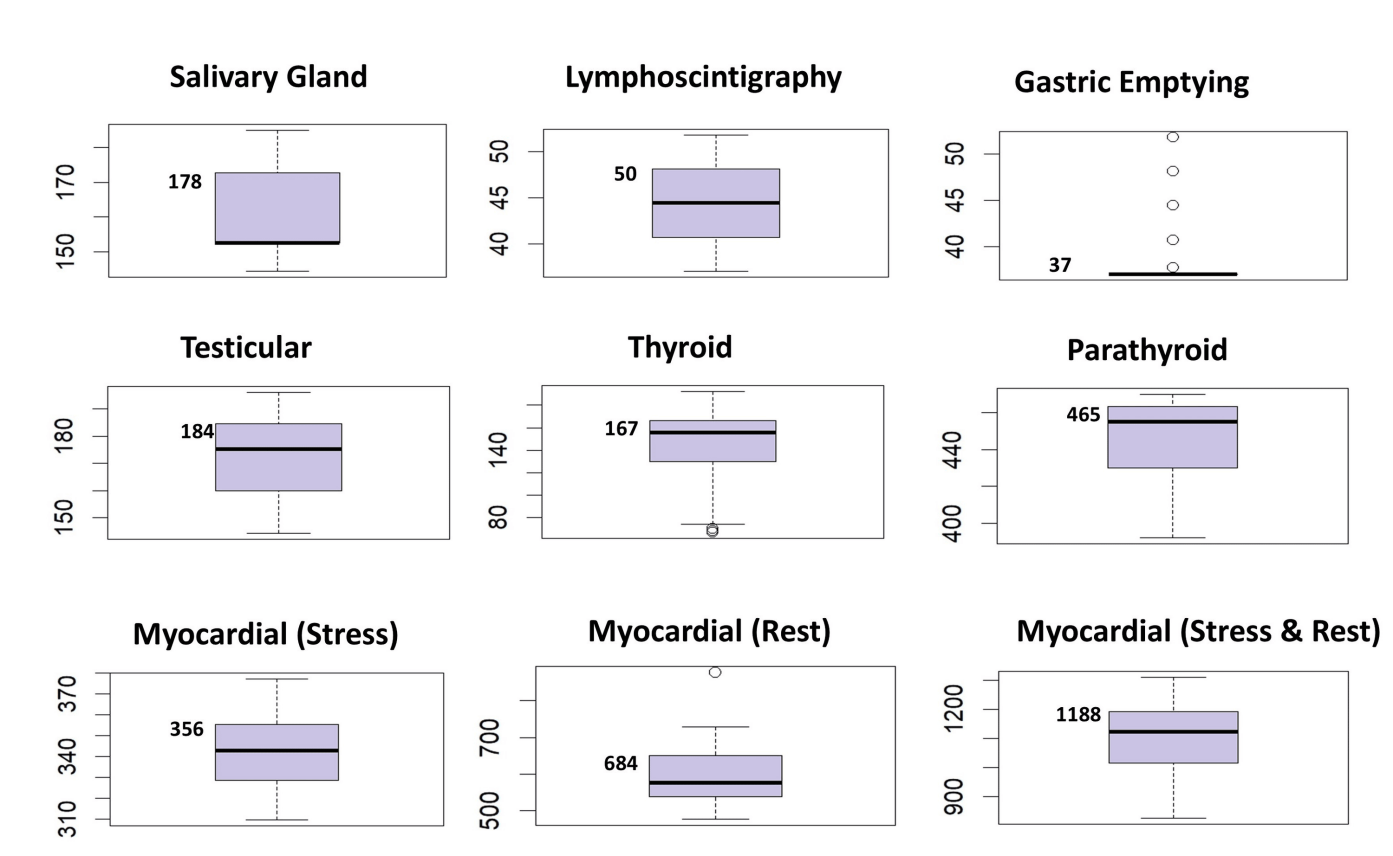
Distribution of institutional DRLs interquartile range showing 75th percentile. DRL: Diagnostic Reference Level.

A box plot displaying the interquartile range and a detailed descriptive statistical summary was created using R software for each imaging procedure (Appendices 1-2). The plot shows data distribution, including median values, the spread of the data within the interquartile range, and outliers. The 75th percentile DRL, indicating the upper quartile of the data, is also prominently shown in Figures 1-2, thus offering a clear visual representation of the variation across the samples.

**Figure 3:**
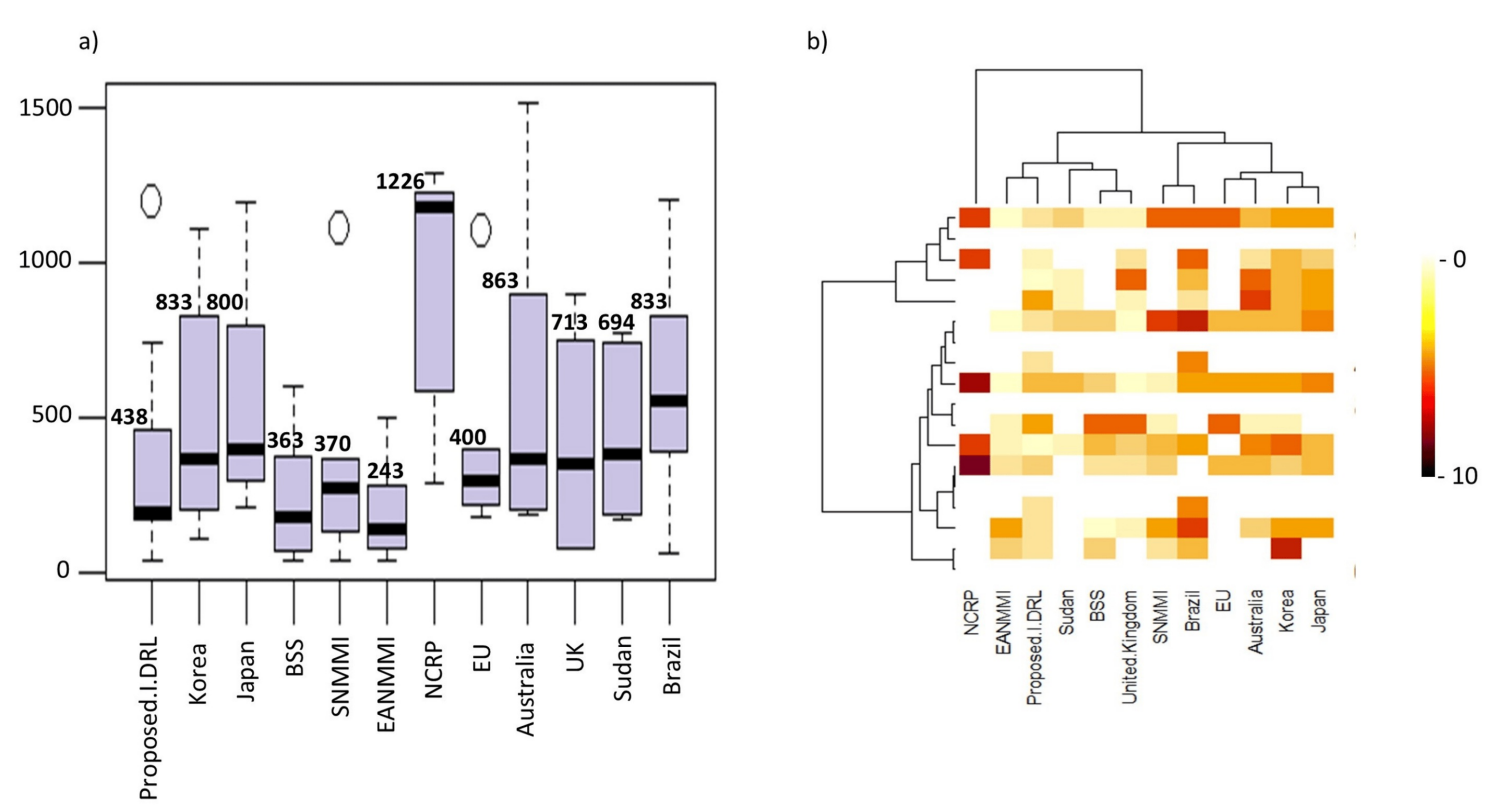
a) Box plot representation of institutional DRLs with international organizations and countries, showing the 75th percentile interquartile range. b) Heat map representing the overall DRL pattern, where a darker region indicates a higher dose pattern in which Institutional DRL (this study) matches with EANMMI, Sudan, BSS, and the United Kingdom, forming one group with similar DRLs, while SNMMI, Brazil, the EU, Australia, Korea, and Japan formed another. The NCRP society remained distinct. DRL: Diagnostic Reference Level; EANMMI: European Association of Nuclear Medicine Medical Internal Radiation Dose; BSS: Basic Safety Standards; SNMMI: Society of Nuclear Medicine and Molecular Imaging; EU: European Union; NCRP: National Council on Radiation Protection and Measurements.

In Table 3, the study compared the institutional DRLs in India with those from international organizations, including Basic Safety Standards (BSS), Society of Nuclear Medicine and Molecular Imaging (SNMMI), European Association of Nuclear Medicine Medical Internal Radiation Dose (EANMMI), National Council on Radiation Protection and Measurements (NCRP), and the European Union (EU). Institutional DRLs were also compared with those from Asian countries (Table 4), African countries (Table 5), European countries, and Australia (Table 6). To visualize the comparison of DRLs across different countries and organizations, the study utilized both a heatmap and a boxplot (Figure 3).

**Table 3:** Comparison of institutional DRL with international organizations. DRL: Diagnostic Reference Level; I-DRL: Institutional Diagnostic Reference Levels; EU: European Union; NCRP: National Council on Radiation Protection and Measurements; EANMMI: European Association of Nuclear Medicine Medical Internal Radiation Dose; SNMMI: Society of Nuclear Medicine and Molecular Imaging; BSS: Basic Safety Standards; 99mTc MDP: 99mTc Methylene Diphosphonate; 99mTc ECD: 99mTc Ethyl Cysteinate Dimer; 99mTc HIDA: 99mTc Hepatobiliary Iminodiacetic Acid.

Imaging Procedure	Radiopharmaceuticals	Proposed I-DRL	BSS [7]	SNMMI [8]	EANMMI [9]	NCRP [10]	EU [4]
Bone Imaging	99mTc MDP	747	600	740-1110	500	848-1185	500-1100
Brain Imaging	99mTc ECD	608				887-1294	
Gastric Emptying Imaging	99mTc Sulphur colloid	37	40	18.5-37	40		
Liver Imaging	99mTc HIDA	177					
Lung Perfusion Imaging	99mTc MAA	197	100	40-150	79	147-226	100-296
Lymphoscintigraphy	99mTc Nano-colloid	50					
Meckel’s Imaging	99mTc pertechnetate	205					
Myocardial Perfusion, stress 1 day	99mTc MIBI	357					
Myocardial Perfusion, rest 1 day	99mTc MIBI	684					
Myocardial Perfusion, stress - rest 1 day	99mTC MIBI	1193					
Renal Imaging	99m Tc EC	156					
Renal Imaging	99m Tc DTPA	170	350	37-370	200	407-587	
Renal Imaging	99m Tc DMSA	181	160	11-110	97	189-289	70-183
Parathyroid Imaging	99mTc MIBI	465					
Thyroid Imaging	99m Tc pertechnetate	166	200	74-370	80		75-222
Salivary Gland Imaging	99m Tc pertechnetate	178	40	185-370	185-370		
Whole body I-131 Low-dose Thyroid Imaging	131 I	327	400	37-185	10-185		90-400
Testicular Imaging	99m Tc pertechnetate	192					

**Table 4:** Comparison of institutional DRL with Asian countries. DRL: Diagnostic Reference Level; I-DRL: Institutional Diagnostic Reference Levels; 99mTc MDP: 99mTc Methylene Diphosphonate; 99mTc ECD: 99mTc Ethyl Cysteinate Dimer; 99mTc HIDA: 99mTc Hepatobiliary Iminodiacetic Acid; 99mTc MAA: 99mTc Macroaggregated Albumin.

Imaging Procedure	Radiopharmaceuticals	Proposed I-DRL	Korea [11]	Japan [12]
Bone Imaging	99mTc MDP	747	925	950
Brain Imaging	99mTc ECD	608	925	800
Gastric Emptying Imaging	99mTc Sulphur colloid	37	111	
Liver Imaging	99mTc HIDA	177		
Lung Perfusion Imaging	99mTc MAA	197	222	260
Lymphoscintigraphy	99mTc Nano-colloid	50		
Meckel’s Imaging	99mTc pertechnetate	205		
Myocardial Perfusion, stress 1 day	99mTc MIBI	357		
Myocardial Perfusion, rest 1 day	99mTc MIBI	684		
Myocardial Perfusion, stress- rest 1 day	99mTC MIBI	1193	555-1110	900-1200
Renal Imaging	99m Tc EC	156		
Renal Imaging	99m Tc DTPA	170	555	400
Renal Imaging	99m Tc DMSA	181	185	210
Parathyroid Imaging	99mTc MIBI	465	740	800
Thyroid Imaging	99m Tc pertechnetate	166	217	300
Salivary Gland Imaging	99m Tc pertechnetate	178	370	370
Whole body I-131 low dose Thyroid Imaging	131 I	327	185	
Testicular Imaging	99m Tc pertechnetate	192		

**Table 5:** Comparison of institutional DRL with African countries. DRL: Diagnostic Reference Level; I-DRL: Institutional Diagnostic Reference Levels; 99mTc MDP: 99mTc Methylene Diphosphonate; 99mTc ECD: 99mTc Ethyl Cysteinate Dimer; 99mTc HIDA: 99mTc Hepatobiliary Iminodiacetic Acid; 99mTc MAA: 99mTc Macroaggregated Albumin.

Imaging Procedure	Radiopharmaceuticals	Proposed I-DRL	Sudan [13]	Brazil [14]
Bone Imaging	99mTc MDP	747	777	1110
Brain Imaging	99mTc ECD	608	-	1203
Gastric Emptying Imaging	99mTc Sulphur colloid	37	-	37-60
Liver Imaging	99mTc HIDA	177		333
Lung Perfusion Imaging	99mTc MAA	197		
Lymphoscintigraphy	99mTc Nano-colloid	50		
Meckel’s Imaging	99mTc pertechnetate	205		555
Myocardial Perfusion, stress 1 day	99mTc MIBI	357		
Myocardial Perfusion, rest 1 day	99mTc MIBI	684		
Myocardial Perfusion, stress- rest 1 day	99mTC MIBI	1193	740	870-925
Renal Imaging	99m Tc EC	156		
Renal Imaging	99m Tc DTPA	170	206	449
Renal Imaging	99m Tc DMSA	181	173	185
Parathyroid Imaging	99mTc MIBI	465	555	740
Thyroid Imaging	99m Tc pertechnetate	166	185	444
Salivary Gland Imaging	99m Tc pertechnetate	178		555
Whole body I-131 low dose Thyroid Imaging	131 I	327		
Testicular Imaging	99m Tc pertechnetate	192		

**Table 6:** Comparison of institutional DRL with European countries and Australia. DRL: Diagnostic Reference Level; I-DRL: Institutional Diagnostic Reference Levels; 99mTc MDP: 99mTc Methylene Diphosphonate; 99mTc ECD: 99mTc Ethyl Cysteinate Dimer; 99mTc HIDA: 99mTc Hepatobiliary Iminodiacetic Acid; 99mTc MAA: 99mTc Macroaggregated Albumin.

Imaging Procedure	Radiopharmaceuticals	Proposed I-DRL	Australia [15]	United Kingdom [16]
Bone Imaging	99mTc MDP	747	920	600
Brain Imaging	99mTc ECD	608	750	750
Gastric Emptying Imaging	99mTc Sulphur colloid	37		
Liver Imaging	99mTc HIDA	177		
Lung Perfusion Imaging	99mTc MAA	197	240	100
Lymphoscintigraphy	99mTc Nano-colloid	50		
Meckel’s Imaging	99mTc pertechnetate	205		
Myocardial Perfusion, stress 1 day	99mTc MIBI	357		
Myocardial Perfusion, rest 1 day	99mTc MIBI	684		
Myocardial Perfusion, stress- rest 1 day	99mTC MIBI	1193	620-1520	800
Renal Imaging	99m Tc EC	156		
Renal Imaging	99m Tc DTPA	170	500	300
Renal Imaging	99m Tc DMSA	181	200	80
Parathyroid Imaging	99mTc MIBI	465	900	900
Thyroid Imaging	99m Tc pertechnetate	166	215	80
Salivary Gland Imaging	99m Tc pertechnetate	178	200	80
Whole body I-131 Low-dose Thyroid Imaging	131 I	327	185	400
Testicular Imaging	99m Tc pertechnetate	192		

## Discussion

In nuclear medicine, the quality of diagnostic outcomes is closely tied to the administered activity of radiopharmaceuticals, which directly impacts both image quality and patient radiation dose. Administered activity varies depending on the type of single-photon emission procedure and the radiopharmaceutical used. Different procedures, such as bone scans with 99mTc-MDP or myocardial perfusion imaging with 99mTc-MIBI, require tailored doses to produce clear images of specific organs or systems. Each radiopharmaceutical has unique properties that determine the optimal dose to ensure sufficient target uptake while minimizing radiation to non-target tissues. Balancing image quality with radiation safety is crucial, and this is guided by the 'as low as reasonably achievable' (ALARA) principle [17], which aims to minimize exposure without compromising diagnostic efficacy. DRLs are used to standardize and optimize dose patterns, ensuring patient safety while maintaining high diagnostic standards across various nuclear medicine procedures.

This study marks the first attempt to standardize dose patterns for nuclear medicine SPECT procedures at a national level in a tertiary care setting in India. Such standardization is vital for optimizing the administered activity levels in various nuclear medicine techniques, ultimately aiding in the reduction and monitoring of dose patterns. Comparing DRLs from this study with international standards is essential, as differences in dose levels could affect patient health.

DRLs were established at the institutional level for various SPECT nuclear medicine modalities and compared with those from other countries and organizations. Following ICRP guidelines [5], the 75th percentile was used to determine these DRLs. The data, including the mean, median, interquartile range, and DRL values for the administered activity, are presented in Table 2, with comparisons illustrated using box plots (Figures 1-2).

The study revealed the highest dose levels in 99mTc MIBI (methoxy isobutyl isonitrile) stress and rest examinations (1193 MBq), followed by 99mTc MDP (747 MBq) and 99mTc ECD (608 MBq). These procedures require higher administered activities to ensure sufficient radiopharmaceutical uptake in large, metabolically active areas (e.g., the heart, bones, or brain). These procedures often target tissues or organs with significant blood flow or metabolic activity, where a higher dose is necessary to produce clear, high-quality images. For example, myocardial perfusion imaging (MIBI) requires high doses to distinguish subtle differences in blood flow between rest and stress conditions in the heart [18]. Bone imaging was the most common procedure performed at this institute, while myocardial perfusion [19] is notable in Iran. On the other hand, procedures like 99mTc gastric emptying (37 MBq) and 99mTc lymphoscintigraphy (50 MBq) involve organs or processes with lower radiopharmaceutical uptake or slower movement through the body. These procedures require less radiopharmaceutical activity because the diagnostic objective can be achieved with lower radiation exposure, given that the stomach and lymphatic system need only minimal tracer amounts to provide clear imaging. Additionally, these areas often involve slower physiological processes, meaning that lower doses are sufficient to produce diagnostic results over time. Procedures such as liver, renal, and parathyroid imaging fall between these extremes, requiring moderate dose levels. These organs are not as metabolically active as the heart or brain but still need a sufficient dose for clear imaging, especially in assessing function or abnormalities. Thus, the variation in dose levels reflects the need to balance image quality with radiation safety, tailoring the administered activity based on the organ's characteristics, the radiopharmaceutical behavior, and the specific diagnostic requirements of the procedure [20].

A heatmap and boxplot were used to compare DRLs across various countries (Figure 3). The boxplot showed the DRL distribution for each country, highlighting the interquartile range with the 75th percentile for comparison. The heatmap used darker colors to represent higher DRL values, offering a visual comparison. This analysis found that the DRLs from the current study align closely with those of the EANMMI society. Additionally, the NCRP society's DRLs were found to be unique. Japan and Korea displayed similar DRL patterns, as did Australia and the EU. It has been previously demonstrated that Korea adheres to ICRP guidelines [21], and it was found that Japan adheres to SNMMI and EU guidelines [22]. Brazil’s DRLs matched those of SNMMI, while the United Kingdom and BSS showed similarities, with Sudan partially aligning with these groups.

Overall, the study identified two major clusters: India (this study), EANMMI, Sudan, BSS, and the United Kingdom formed one group with similar DRLs, while SNMMI, Brazil, the EU, Australia, Korea, and Japan formed another. The NCRP society remained distinct in a separate cluster (Figure 3).

The EANMMI sets guidelines and standards for nuclear medicine procedures across Europe, emphasizing the safe and effective use of radiopharmaceuticals for both diagnostic and therapeutic purposes. Their recommendations focus on DRLs, radiation dose optimization, and patient safety, aligning with international standards such as those from the IAEA and the ICRP. The inclusion of EANMMI guidelines ensures accurate dosimetry calculations, helping optimize patient radiation exposure while maintaining high diagnostic quality, particularly in SPECT and positron emission tomography (PET) procedures. The alignment of the present study’s DRLs with those of the EANMMI society indicates adherence to European practices, which are recognized for their rigorous standards of radiation protection and patient care [22].

On the other hand, the NCRP sets DRLs specific to United States (US) practices, focusing on optimizing radiopharmaceutical doses for procedures like SPECT and PET while maintaining high diagnostic standards. The uniqueness of the NCRP DRLs can be attributed to the US's specific radiation safety culture, the use of various radiopharmaceuticals, and differences in procedural protocols, leading to its overall differences when compared with international standards like those from the EANM or IAEA [23].

The SNMMI, another renowned US-based organization, also establishes DRLs and guidelines to advance nuclear medicine and molecular imaging. Like the NCRP, the SNMMI prioritizes lowering patient radiation doses while ensuring the validity of diagnostic procedures involving radiopharmaceuticals [8].

Lastly, the BSS, developed by organizations like the IAEA and the WHO, provides globally recognized guidelines that target justification, optimization, and dose limitation to ensure safe radiation use in nuclear medicine [24]. By adhering to these principles, the study contributes to translational efforts to optimize radiation safety and align closely with EANMMI standards. These international comparisons of DRLs emphasize the study’s commitment to maintaining high levels of patient safety and care across various nuclear medicine practices globally.

The limitations of this study include that it was carried out in a single tertiary care setting, which may not represent the variations present in different healthcare settings at the national level. Besides comparisons made with international DRLs, differences in patient demographics, clinical practices, and equipment may influence the observed dose patterns. Institutional DRLs will function as practical models that aid in the development of national DRLs.

## Conclusions

This study proposed the first institutional DRLs for 18 SPECT nuclear medicine studies in India at the tertiary care level. This research represents a significant step toward establishing standardized DRLs for nuclear medicine procedures in India, facilitating a standard for radiation dose optimization in a tertiary care setting. By analyzing 18 common nuclear medicine procedures and comparing the DRLs with international standards from societies such as EANMMI, NCRP, and SNMMI, the research highlights key radiation dose variations and aligns India’s practices with global radiation safety frameworks. The findings emphasize the need for continued dose monitoring and optimization, particularly for high-dose procedures like 99mTc MIBI stress and rest examinations, while reinforcing patient safety in lower-dose procedures such as 99mTc gastric emptying and lymphoscintigraphy. The establishment of these DRLs, aligned with international guidelines from the EANMMI, IAEA, and ICRP, provides a robust foundation for future dose regulation, ensuring safe and effective nuclear medicine practices in India and supporting the global discourse on radiation protection.

## Appendices

Appendix 1

**Table 7:** Descriptive statistics of various imaging procedures.

Imaging Procedure	Min.	Q1	Median	Mean	Q3	Max.
Bone	268.2	540.2	703.0	639.2	740.0	939.8
Gastric emptying	37.00	37.00	37.00	38.00	37.00	51.80
EC	88.8	133.4	148.2	142.3	156.5	181.3
DTPA	85.1	146.8	160.0	155.6	170.2	188.7
Brain	355.2	496.7	573.5	528.2	605.0	610.5
DMSA	77.7	166.8	170.6	159.7	181.3	188.7
HIDA	144.3	155.8	170.2	167.3	176.9	188.7
I-131	159.1	185.0	207.2	247.9	306.2	410.7
Lymphoscintigraphy	37.0	40.7	44.4	44.4	48.1	51.8
MAA	151.7	161.1	174.3	175.9	192.4	200.2
Meckels	181.3	181.3	192.4	193.1	199.8	210.9
Rest	476.9	538.4	577.2	601.3	651.2	876.9
Parathyroid	392.2	429.9	455.1	444.2	463.2	469.9
Salivary Gland	144.3	152.4	152.4	161.4	172.8	185.0
Thyroid	66.6	129.5	155.4	146.9	166.5	192.4
Testicular	144.3	159.8	175.4	172.1	184.4	196.1
Stress	309.7	328.7	343.0	343.9	355.6	377.4
Stress and rest	268.2	540.2	703.0	639.2	740.0	939.8

Appendix 2 

**Table 8:** Descriptive statistics of various countries and organizations' DRL. DRL: Diagnostic reference level; I-DRL: Institutional Diagnostic Reference Levels; BSS: Basic Safety Standards; SNMMI: Society of Nuclear Medicine and Molecular Imaging; EANMMI: European Association of Nuclear Medicine Medical Internal Radiation Dose; NCRP: National Council on Radiation Protection and Measurements; EU: European Union.

Imaging Procedure	Min.	Q1	Median	Mean	Q3	Max.
Proposed I-DRL	37.0	171.8	194.5	338.3	438.0	1193.0
Korea	111.0	201.0	370.0	504.1	832.5	1110.0
Japan	210.0	300.0	400.0	587.8	800.0	1200.0
Australia	185.0	203.8	370.0	563.0	862.5	1520.0
United Kingdom	80.0	85.0	350.0	409.0	712.5	900.0
Sudan	173.0	190.2	380.5	439.3	693.8	777.0
Brazil	60.0	388.5	555.0	596.3	832.5	1203.0
BSS	40.0	85.0	180.0	236.2	362.5	600.0
SNMMI	37.0	140.0	277.5	337.8	370.0	1110.0
EANMMI	40.00	79.75	141.00	193.88	242.50	500.00
NCRP	289.0	587.0	1185.0	916.2	1226.0	1294.0
EU	183.0	222.0	296.0	440.2	400.0	1100.0

## References

[1] Bethesda M (2009). NCRP Report No. 160: Ionizing Radiation Exposure of the Population of the United States. J Radiol Prot.

[2] Do KH (2016). General principles of radiation protection in fields of diagnostic medical exposure. J Korean Med Sci.

[3] Cho SG, Kim J, Song HC (2017). Radiation safety in nuclear medicine procedures. Nucl Med Mol Imaging.

[4] (2021). European Commission Radiation Protection N° 180. https://www.eurosafeimaging.org/wp/wp-content/uploads/2015/05/Radiation-protection-180-part2.pdf.

[5] Vañó E, Miller DL, Martin CJ (2017). ICRP Publication 135: diagnostic reference levels in medical imaging. Ann ICRP.

[6] Gupta MM (1998). Nuclear medicine practice and radiation doses to patients in India: Part II. Risk related radiation doses
to patients. ICMR Bulletin.

[7] International Atomic Energy Agency (IAEA) (1996). International basic safety standards for protection against ionizing radiation and for the safety of radiation sources. Vienna, Austria: IAEA [Internet.

[8] Alessio AM, Farrell MB, Fahey FH (2015). Role of reference levels in nuclear medicine: a report of the SNMMI dose optimization task force. J Nucl Med.

[9] (2021). European Association of Nuclear Medicine and Molecular Imaging - EANMMI. Guidelines. https://www.eanm.org/publications/guidelines/.

[10] NCRP NCRP (2012). Report # 172: Reference Levels and Archievable Doses in Medical and Dental Imaging: Recommendations for Applications in the United States [Internet]. National Council on Radiation Protection and Measurements. https://ncrponline.org/shop/reports/report-no-172-reference-levels-and-achievable-doses-in-medical-and-dental-imaging-recommendations-for-the-united-states-2012/.

[11] Song HC, Na MH, Kim J, Cho SG, Park JK, Kang KW (2019). Diagnostic reference levels for adult nuclear medicine imaging established from the National Survey in Korea. Nucl Med Mol Imaging.

[12] Watanabe H, Ishii K, Hosono M (2016). Report of a nationwide survey on actual administered radioactivities of radiopharmaceuticals for diagnostic reference levels in Japan. Ann Nucl Med.

[13] Ali W, Elawad R, Ibrahim M (2016). Establishment of dose reference levels for nuclear medicine in Sudan. Open J Radiol.

[14] Willegaignon J, Braga LF, Sapienza MT (2016). Diagnostic reference level: an important tool for reducing radiation doses in adult and pediatric nuclear medicine procedures in Brazil. Nucl Med Commun.

[15] (2021). Nuclear Medicine Diagnostic Reference Levels (DRLs). https://www.arpansa.gov.au/sites/default/files/nuclear-medicine-diagnostic-reference-levels.pdf.

[16] Iball GR, Bebbington NA, Burniston M (2017). A national survey of computed tomography doses in hybrid PET-CT and SPECT-CT examinations in the UK. Nucl Med Commun.

[17] Kaplan DJ, Patel JN, Liporace FA, Yoon RS (2016). Intraoperative radiation safety in orthopaedics: a review of the ALARA (as low as reasonably achievable) principle. Patient Saf Surg.

[18] Holder L, Lewis S, Abrames E (2016). Review of SPECT myocardial perfusion imaging. Official J Am Osteopath Coll Radiol.

[19] Niksirat F, Monfared AS, Deevband MR, Amiri M, Gholami A (2016). Estimating the population dose from nuclear medicine examinations towards establishing diagnostic reference levels. Indian J Nucl Med.

[20] Wang Z, Ying Z, Bosy-Westphal A (2010). Specific metabolic rates of major organs and tissues across adulthood: evaluation by mechanistic model of resting energy expenditure. Am J Clin Nutr.

[21] Kim BI (2016). Radiological justification for and optimization of nuclear medicine practices in Korea. J Korean Med Sci.

[22] Del Guerra A, Bardies M, Belcari N (2013). Curriculum for education and training of medical physicists in nuclear medicine: recommendations from the EANM Physics Committee, the EANM Dosimetry Committee and EFOMP. Phys Med.

[23] Brink JA, Miller DL (2015). U.S National Diagnostic Reference Levels: closing the gap. Radiol.

[24] Janžekovič H (2017). Differences between IAEA and EU basic safety standards. NENE.

